# Nutraceutical Value of Yellow- and Red-Fleshed South African Plums (*Prunus salicina* Lindl.): Evaluation of Total Antioxidant Capacity and Phenolic Composition

**DOI:** 10.3390/molecules19033084

**Published:** 2014-03-11

**Authors:** Alet Venter, Elizabeth Joubert, Dalene de Beer

**Affiliations:** 1 Department of Food Science, Stellenbosch University, Private Bag X1, Matieland (Stellenbosch) 7602, South Africa; E-Mail: aletventer4@gmail.com; 2 Post-Harvest and Wine Technology Division, Agricultural Research Council (ARC), Infruitec-Nietvoorbij, Private Bag X5026, Stellenbosch 7599, South Africa; E-Mail: joubertL@arc.agric.za

**Keywords:** antioxidant capacity, HPLC, phenolic compound, plum fruit, *Prunus salicina* Lindl

## Abstract

Ten South African plum cultivars and selections, including yellow- and red-fleshed plums from two harvest seasons were evaluated in terms of nutraceutical value (phenolic composition, total polyphenol content (TPC) and total antioxidant capacity (TAC)) and fruit attributes (colour, fruit mass, *etc.*). Plums were evaluated at two maturity stages, *i.e.*, directly after harvest (unripe) and after a commercial cold storage and ripening regime (ripe). The phenolic composition of South African plums varied greatly, both qualitatively and quantitatively, between cultivars and selections. Neochlorogenic acid, cyanidin-3-*O*-glucoside and quercetin-3-*O*-glucoside were the predominant hydroxycinnamic acid, anthocyanin and flavonol, respectively, although not present in all plums. The flavan-3-ols, (+)-catechin, (−)-epicatechin and procyanidins B1 and B2, were present in all plums. Red-fleshed plums tended to display higher TAC and TPC than yellow-fleshed plums. The flavan-3-ol content was highly correlated with TAC. The effect of harvest season was cultivar-dependent, but cultivar differences were not obscured. In terms of maturity stage, the ripe fruits tended to contain higher levels of anthocyanins and some flavonol compounds, although the TPC and TAC were not affected in most cases. South African plums, especially the red-fleshed selections PR04-32 and PR04-35, were shown to provide generally high TAC and TPC compared to literature values.

## 1. Introduction

Plums grown in South Africa for the fresh fruit market are classified as Japanese plums (*Prunus salicina* Lindl.). Japanese plums differ from European plums (*Prunus domestica* L.) in various aspects such as size and composition of volatile compounds, sugars, organic acids and phenolic compounds [1,2]. Most of the plums produced in South Africa are exported (*ca*. 74% of the total production), with Europe and the United Kingdom being the major markets [3]. Plums are one of the fruit types known to generally contain high concentrations of phenolic compounds [4,5]. Plum phenolic compounds have been shown to exhibit several health benefits, including antiproliferative activity against breast cancer cell lines [6], immunostimulatory activity [7], hypoglycaemic activity [8], mitigation of age-related cognitive decline [9] and chemopreventive activity against carcinogens [10]. The antioxidant activity of phenolic compounds is believed to contribute to the health benefits of plums [11]. Due to the nutritional and nutraceutical value of plums, it has been suggested as an important fruit to incorporate into the diet [5]. Interest in the nutraceutical value of foods has guided plant breeders to select genotypes for further evaluation based on phenolic content and antioxidant activity [12].

Attributes such as phenolic composition, total antioxidant capacity (TAC), nutrient content and physical aspects (colour, fruit mass, *etc.*) differ between plum cultivars [12,13,14,15] and at different stages of maturity [16,17,18]. These factors are also affected by pre- and post-harvest elements such as the environment, climate, cold storage parameters, *etc.* In South Africa, plums are harvested at a pre-climacteric stage (termed commercial ripeness which is defined per cultivar) and subjected to cold storage prior to marketing. This enables transport of fruits to markets in other countries or increasing the marketable period of the fruits. Prior to retail the fruits are allowed to ripen by increasing the storage temperature for a few days. An increase in the anthocyanin content during ripening of plum fruits is visible in red-skinned cultivars with the skin colour changing from green to predominantly red [19]. Harvest season may also affect the phenolic composition of plums [20]. Such changes in phenolic composition of fruits are likely to affect their TAC. Currently no information is available on the effect of cold storage and subsequent ripening on the phenolic composition and TAC of South African plums.

The aim of this study was to compare the nutraceutical value (phenolic composition, total polyphenol content (TPC) and TAC) of yellow- and red-fleshed plum cultivars and selections from South Africa, and evaluate the effect of maturity stage and harvest season on these factors. Fruit attributes (skin and flesh colour, fruit mass, total dissolved solids, titratable acidity, *etc.*) were also evaluated to characterise plum cultivars and selections.

## 2. Results and Discussion

Plum fruit (*Prunus salicina* Lindl.) from a range of yellow- and red-fleshed cultivars and selections were harvested during the summer of 2010/2011 (December 2010 to February 2011; first harvest season) and 2011/2012 (December 2011 to February 2012; second harvest season) (Table 1). All evaluated cultivars and selections originated from and were grown in South Africa. Among the cultivars and selections evaluated, Laetitia, African Delight and Sapphire are among the top four cultivars produced in South Africa in terms of area under production [3], while Ruby Crunch is a newly released red-fleshed cultivar. Sun Breeze was investigated as an example of a plum cultivar with both yellow skin and flesh. A number of red-fleshed plum selections currently undergoing evaluation were also included as this type of plum is currently a focus of the ARC Infruitec-Nietvoorbij plum breeding programme (personal communication, C. Smith, Cultivar Development Division, ARC Infruitec-Nietvoorbij, Stellenbosch, South Africa). As all cultivars and selections were not available for harvesting during the first harvest season, data obtained for fruit harvested during the second harvest season will be used to illustrate differences between cultivars and selections, as well as the effect of maturity stage. Data obtained during the first harvest season is given in the Supplementary Information (Table S1–S3). Data from cultivars and selections available in both harvest seasons will be used to illustrate the effect of harvest season. Typical chromatograms for each cultivar and selection are shown by Venter *et al.* [21].

**Table 1:** Skin and flesh colour and harvest dates of South African plum (*Prunus salicina* Lindl.) cultivars and selections.

Cultivar/Selection	Photo	Skin Colour (Ripe)	Flesh Colour (Ripe)	Harvest Date	Harvest Date
				(First Season)	(Second Season)
Sun Breeze	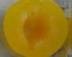	Yellow	Yellow	1 February 2011	14 February 2012
African Delight	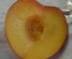	Red	Yellow	22 February 2011	12 February 2012
Laetitia	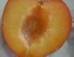	Red	Yellow	8 February 2011	8 February 2012
Ruby Crunch	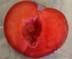	Red	Red	- ^a^	31 January 2012
Ruby Red	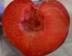	Red	Red	4 January 2011	3 January 2012
Sapphire	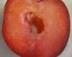	Red	Red	14 December 2010	13 December 2011
PR03-34 ^b^	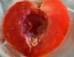	Red	Red	21 December 2010	20 December 2011
PR04-19 ^b^	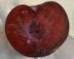	Red	Red	- ^a^	13 December 2011
PR04-32 ^b,c^	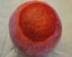	Red	Red	25 January 2011	17 January 2012
PR04-35 ^b^	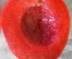	Red	Red	- ^a^	20 December 2011

^a^ Cultivar/selection not available for harvesting in the first season; ^b^ selection numbers for plums without cultivar names currently in evaluation trials; ^c^ photo indicates area where flesh colour was measured after removal of skin.

### 2.1. Comparison of Cultivars and Effect of Maturity Stage

**Figure 1:**
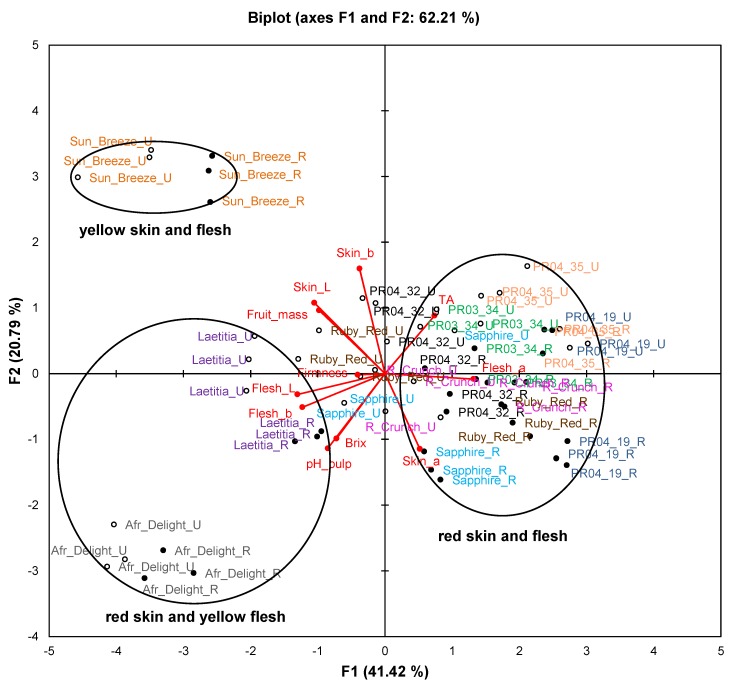
Principal component biplot of fruit attributes of unripe and ripe fruits from South African plum (*Prunus salicina* Lindl.) cultivars and selections of the second harvest season.

*Abbreviations:* R, ripe fruit (closed circles); TA, titratable acidity; TDS, total dissolved solids; U, unripe fruit (open circles).

**Figure 2:**
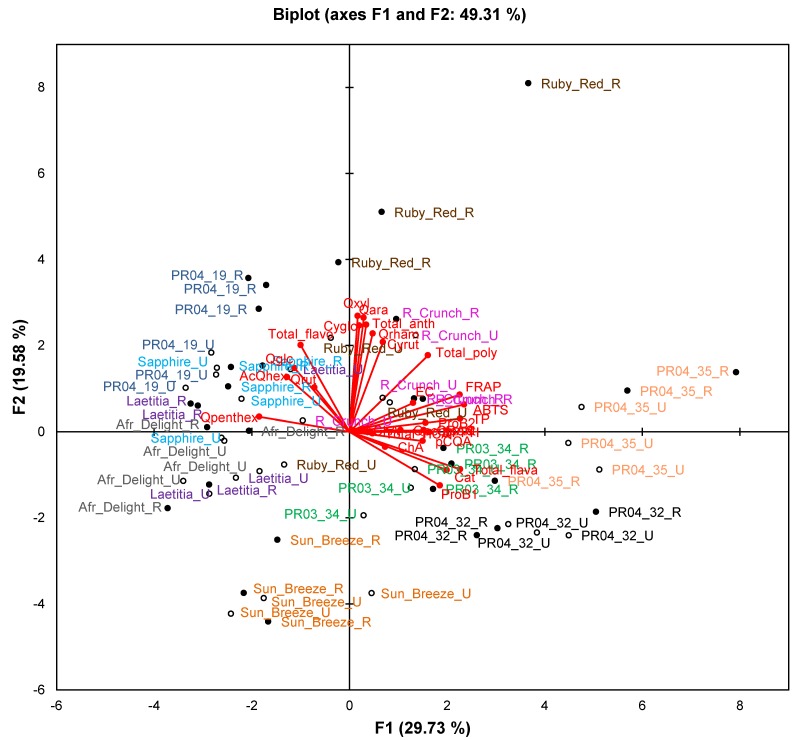
Principal component biplot of phenolic composition and total antioxidant capacity of unripe and ripe fruits from South African plum (*Prunus salicina* Lindl.) cultivars and selections of the second harvest season.

*Abbreviations:* AcQhex, quercetin-acetylhexoside; antho, anthocyanin; Cat, (+)-catechin; ChA, chlorogenic acid; Cyrut, cyanidin-3-*O*-rutinoside; Cyglc, cyanidin-3-*O*-glucoside; EC, (−)-epicatechin; flavo, flavonol; flava, flavan-3-ol; HCA, hydroxycinnamic acids; NeoChA, neochlorogenic acid; pCoumaroylq, 3-*O*-*p*-coumaroylquinic acid; ProB1, procyanidin B1; ProB2, procyanidin B2; Qara, quercetin-3-*O*-arabinoside; Qglc, quercetin-3-*O*-glucoside; Qpenthex, quercetin pentosyl-hexoside; Qpentpent, quercetin pentosyl-pentoside; Qrham, quercetin-3-*O*-rhamnoside; Qrut, quercetin-3-*O*-rutinoside; Qxyl, quercetin-3-*O*-xyloside; R, ripe fruit (closed circles); U, unripe fruit (open circles).

Principal Component Analysis (PCA) biplots were compiled to evaluate the association between samples, fruit attributes, TAC and phenolic composition (Figure 1 and Figure 2). The correlations between variables were also statistically evaluated using Pearson’s correlation coefficients (R values) (selected R values shown in Table 2). ANOVA was performed on the average values of five pooled fruits from each of three trees (Table 3, Table 4 and Table 5).

**Table 2:** Correlations (*r*) between antioxidant capacity and selected parameters for ripe fruit from the second harvest season.

Variables	ABTS^•+^^a^	DPPH^•^^b^	FRAP ^c^	ORAC ^d^	TPC ^e^
**Fruit mass**	−0.521 **	−0.373 *	−0.545 **	0.025 ^ns^	−0.385 *
Flesh a*	0.807 ***	0.396 *	0.740 ***	0.403 *	0.606 ***
**Total anthocyanins**	0.370 *	0.067 ^ns^	0.397 *	0.052 ^ns^	0.149 ^ns^
**Total hydroxycinnamic acids**	0.132 ^ns^	0.094 ^ns^	0.252 ^ns^	0.102 ^ns^	0.382 *
**Total flavan-3-ols**	0.712 ***	0.542 **	0.642 ***	0.577 **	0.723 ***
**Total flavonols **	−0.189 ^ns^	0.012 ^ns^	−0.057 ^ns^	−0.095 ^ns^	−0.097 ^ns^
**TPC ^e^**	0.914 ***	0.724 ***	0.950 ***	0.482 **	-

* *p* < 0.05; ** *p* < 0.01; *** *p* < 0.001; ^ns^*p* ≥ 0.05; ^a^ total antioxidant capacity (TAC) measured using the ABTS^•+^ scavenging assay; ^b^ TAC measured using the DPPH scavenging assay; ^c^ TAC measured using the ferric reducing antioxidant power assay; ^d^ TAC measured using the oxygen radical absorbance capacity assay; ^e^ total polyphenol content measured using the Folin-Ciocalteau assay.

Figure 1 represents fruit attributes (fruit mass, total dissolved solids (TDS), titratable acidity (TA), pH, as well as skin and flesh colour) for ripe and unripe fruit collected during the second harvest season. The biplot explains 62.2% of the variation. Samples of Sun Breeze and African Delight formed distinct clusters without any overlap with others, while samples of the other cultivars and selections formed overlapping clusters. Furthermore, observations for ripe and unripe fruit were clearly separated for all cultivars and selections.

The cultivars and selections were mainly separated on the PCA biplot (Figure 1) along F1 based on their flesh colour and along F2 based on their skin colour. In addition, some observations of unripe fruits from red-fleshed cultivars and selections (notably Ruby Red and Sapphire) were situated in the centre of the plot corresponding to cultivars and selections where flesh colour changed from yellow to red during fruit ripening. A pronounced increase (*p* < 0.05) in the flesh a*-value of Ruby Red occurred as a result of ripening, while a more modest increase (*p* < 0.05) was observed for Ruby Crunch and Sapphire (Table 3). Skin and flesh L*-values decreased significantly (*p* < 0.05) from unripe to ripe fruits for most cultivars and selections, indicating darker colour (Table 3).

The PCA biplot based on phenolic composition and TAC of cultivars/selections (Figure 2) only explains 49.3% of the variation. Observations for selections PR04-32 and PR04-35 formed two separate clusters in the right-hand quadrants of the plot, while observations for Sun Breeze formed a separate cluster in the lower quadrants (mainly lower left) of the plot. Other cultivars and selections generally showed overlapping clusters, except ripe Ruby Red and PR04-19 samples that were also separated from other samples. The TAC measured in the ABTS^•+^, DPPH^•^, FRAP and ORAC assays and TPC measured in the Folin-Ciocalteau assay associated with each other and with selections PR04-32 and PR04-35. These two selections generally had the highest or among the highest TAC and TPC (Table 4).

**Table 3:** Fruit attributes of unripe and ripe fruits from South African plum (*Prunus salicina* Lindl.) cultivars and selections of the second harvest season ^a^.

Cultivar/Selection	Stage of Maturity	Fruit Mass (g)	Firmness (kg)	pH	TA ^b^	TDS (°Brix) ^c^	Skin a* ^d^	Skin b* ^d^	Skin L* ^d^	Flesh a* ^d^	Flesh b* ^d^	Flesh L* ^d^
Sun Breeze	Unripe	111.3 a	10.0 ab	3.91 cd	23.9 efg	15.0 ef	−8.7 l	25.5 a	58.7 a	−3.0 j	21.3 d	47.5 c
	Ripe	105.9 a	3.0 hi	3.97 bc	24.5 defg	14.3 gh	−0.5 k	27.3 a	58.4 a	−0.3 ij	17.6 ef	41.7 e
African Delight	Unripe	80.7 cd	11.1 a	4.19 a	14.9 h	21.0 a	21.9 c	4.0 g	48.9 b	1.6 hi	29.2 a	53.9 a
	Ripe	77.8 cde	7.9 cde	4.22 a	12.5 h	19.3 b	18.8 e	−0.04 ij	42.8 de	2.3 hi	27.4 b	51.5 b
Laetitia	Unripe	85.1 bc	8.0 cd	3.93 bcd	30.8 c	14.2 gh	22.1 c	10.4 e	49.7 b	2.1 hi	26.5 b	50.5 b
	Ripe	82.9 bc	2.7 i	3.99 bc	22.9 fg	13.7 h	21.8 cd	1.3 hi	41.4 ef	4.2 gh	23.0 c	48.3 c
Ruby Crunch	Unripe	83.8 bc	9.0 bc	3.86 de	29.8 c	15.1 ef	12.4 j	−1.5 jk	37.2 h	14.8 e	17.6 ef	37.7 f
	Ripe	81.1 bcd	4.4 gh	3.91 cd	30.7 c	14.0 gh	12.0 j	−2.6 k	33.3 ij	22.0 ab	13.1 h	32.5 gh
Ruby Red	Unripe	84.1 bc	7.9 cd	3.86 def	37.8 b	17.2 c	19.3 e	11.9 de	42.0 ef	7.0 g	22.9 c	44.8 d
	Ripe	78.1 cde	5.4 fg	3.84 def	30.4 c	16.4 d	15.0 hi	−1.7 jk	29.7 kl	20.5 abc	12.2 hi	30.5 ij
Sapphire	Unripe	88.6 b	3.4 hi	3.76 ef	29.6 cd	15.2 ef	13.6 ij	1.7 ghi	31.7 jk	10.6 f	21.4 d	43.5 d
	Ripe	78.5 cde	2.4 i	4.02 b	20.4 g	14.1 gh	19.6 de	3.2 gh	28.0 lm	15.6 de	18.5 e	38.1 f
PR03-34 ^e^	Unripe	82.2 bcd	3.6 hi	3.84 def	30.1 c	16.1 d	16.2 fgh	14.3 cd	44.2 cd	21.0 ab	14.6 g	33.8 g
	Ripe	75.0 def	2.9 hi	3.93 bcd	22.5 fg	14.6 fg	25.5 b	14.5 c	42.3 de	20.6 abc	11.6 i	29.1 j
PR04-19 ^e^	Unripe	58.4 g	7.4 cde	3.75 f	47.1 a	15.4 e	15.8 ghi	6.9 f	34.5 i	21.2 ab	12.1 hi	31.9 hi
	Ripe	50.6 h	6.6 def	3.98 bc	24.4 defg	14.5 fg	15.2 hi	2.8 gh	27.5 m	15.9 de	7.2 j	25.9 k
PR04-32 ^e^	Unripe	78.2 cde	8.5 bc	3.92 bcd	27.6 cdef	12.8 i	18.4 ef	20.2 b	44.8 c	18.0 cd	21.4 d	40.5 e
	Ripe	75.0 def	6.3 ef	3.98 bc	20.7 g	12.3 i	29.2 a	15.1 c	40.2 fg	19.6 bc	16.6 f	36.6 f
PR04-35 ^e^	Unripe	71.0 ef	8.8 bc	3.77 ef	36.7 b	12.8 i	18.0 efg	13.6 cd	43.4 cde	20.4 abc	12.7 hi	33.9 g
	Ripe	68.3 f	8.9 bc	3.79 ef	28.8 cde	11.4 j	29.2 a	13.2 cd	39.1 gh	23.2 a	12.0 hi	31.6 hi

^a^ Values represent averages of plums from three trees (5 fruits per tree); different letters in the same column indicate a statistically significant difference (*p* < 0.05); ^b^ titratable acidity in g malic acid/kg fresh weight; ^c^ total dissolved solids expressed as °Brix; ^d^ skin and flesh colour values using the CIELab scale; ^e^ selection numbers for plums without cultivar names currently in evaluation trials.

**Table 4:** Total polyphenol content (TPC) ^a^ and total antioxidant capacity (TAC) ^b^ of unripe and ripe fruits from South African plum (*Prunus salicina* Lindl.) cultivars and selections of the second harvest season ^c^.

Cultivar/Selection	Stage of Maturity	TPC ^a^	ABTS^•+ b,d^	DPPH^• b,e^	FRAP ^b,f^	ORAC ^b,g^
Sun Breeze	Unripe	2.56 ghi	23.4 e	12.6 ghij	9.5 gh	40.2 defg
	Ripe	2.32 hi	21.9 e	12.5 hij	9.0 h	41.4 cdef
African Delight	Unripe	2.62 efghi	24.8 e	16.4 abc	10.6 efg	37.5 defg
	Ripe	2.65 efghi	23.8 e	16.4 bc	10.5 efg	38.4 defg
Laetitia	Unripe	2.76 defgh	24.5 e	13.1 fghij	10.4 efg	36.7 defg
	Ripe	2.58 fghi	23.0 e	12.4 hij	9.8 gh	35.4 defg
Ruby Crunch	Unripe	2.68 efgh	29.3 cd	13.9 efgh	11.7 cde	42.5 cde
	Ripe	3.02 cde	32.1 bcd	15.2 cde	12.6 cd	50.5 bc
Ruby Red	Unripe	2.40 ghi	23.2 e	11.4 i	9.5 gh	58.1 ab
	Ripe	2.98 cdef	29.7 cd	14.6 cdef	12.5 cd	55.4 ab
Sapphire	Unripe	2.25 i	25.0 e	12.6 ghij	9.3 gh	32.0 g
	Ripe	2.27 hi	25.0 e	13.3 fghi	9.7 gh	32.6 fg
PR03-34 ^h^	Unripe	2.74 efg	29.3 cd	13.8 efghi	11.3 def	44.2 cd
	Ripe	2.98 defg	33.0 bc	15.5 cde	12.3 cd	42.1 cde
PR04-19 ^h^	Unripe	2.31 hi	25.3 e	12.0 ij	10.0 fgh	31.4 g
	Ripe	2.62 efghi	29.1 d	14.4 defg	11.8 cde	33.7 efg
PR04-32 ^h^	Unripe	3.21 bc	33.6 b	18.2 a	13.0 bc	54.7 ab
	Ripe	3.16 bcd	32.5 bcd	17.7 ab	12.5 cd	62.2 a
PR04-35 ^h^	Unripe	3.48 ab	37.8 a	15.8 cd	14.2 ab	39.1 defg
	Ripe	3.83 a	38.0 a	15.9 bcd	15.2 a	44.2 cd

^a^ TPC in g gallic acid equivalents/kg fresh weight as measured using the Folin-Ciocalteau assay; ^b^ TAC in µmol Trolox equivalents/g fresh weight; ^c^ values represent averages of plums from three trees (5 fruits per tree); different letters in the same column indicate a statistically significant difference (*p* < 0.05); ^d^ TAC measured using the ABTS^•+^ scavenging assay; ^e^ TAC measured using the DPPH^•^ scavenging assay; ^f^ TAC measured using the ferric reducing antioxidant power assay; ^g^ TAC measured using the oxygen radical absorbance capacity assay; ^h^ selection numbers for plums without cultivar names currently in evaluation trials.

Table 5Individual phenolic compound content (mg/kg fresh weight) of unripe and ripe fruits from South African plum (*Prunus salicina* Lindl.) cultivars and selections of the second harvest season ^a^.

**Table:** 

Cultivar/Selection	Stage of Maturity	Cyanidin-3-*O*-glucoside	Cyanidin-3-*O*-rutinoside	Neochlorogenic Acid	Chlorogenic Acid	3-*O-p*-Coumaroyl-quinic Acid	(−)-Epicatechin	(+)-Catechin	Procyanidin B1	Procyanidin B2
Sun Breeze	Unripe	ND i	ND i	224.6 fg	ND c	11.7 c	9.2 ij	81.3 e	216.1 cd	18.7 g
	Ripe	ND i	ND i	212.7 g	ND c	10.7 c	8.4 ij	75.5 ef	204.8 cd	17.0 gh
African Delight	Unripe	29.7 ghi	7.9 hi	376.3 bc	22.2 a	ND d	12.6 i	77.3 ef	121.8 e	11.5 gh
	Ripe	69.3 ef	13.1 hi	356.0 c	21.7 a	ND d	5.0 j	67.0 efg	104.4 efg	9.9 h
Laetitia	Unripe	15.1 hi	7.0 hi	434.8 a	ND c	19.3 bc	12.7 i	80.2 e	115.1 ef	10.6 gh
	Ripe	52.5 fg	15.3 h	376.3 cd	ND c	15.1 bc	10.5 ij	65.5 efg	99.8 efg	8.6 h
Ruby Crunch	Unripe	86.3 e	100.0 c	56.6 h	ND c	ND d	42.4 de	136.3 bcd	271.8 b	37.1 e
	Ripe	230.0 b	120.4 b	57.5 h	ND c	ND d	42.4 de	156.9 abc	243.9 bc	34.4 ef
Ruby Red	Unripe	33.1 gh	19.3 gh	409.9 ab	ND c	20.5 bc	59.5 b	64.8 efg	81.2 fg	62.8 b
	Ripe	474.9 a	85.0 c	433.0 a	ND c	23.4 b	57.6 b	56.4 efg	64.9 gh	52.6 c
Sapphire	Unripe	177.4 c	31.3 efg	ND i	ND c	ND d	48.0 cd	61.8 efg	75.8 fgh	53.7 c
	Ripe	231.6 b	56.5 d	ND i	ND c	ND d	51.5 c	60.9 efg	73.5 gh	47.6 cd
PR03-34^ b^	Unripe	189.6 c	48.5 de	237.2 efg	11.6 b	113.8 a	19.0 h	120.5 d	199.6 d	15.6 gh
	Ripe	232.8 b	55.5 d	255.6 def	11.4 b	115.4 a	22.4 h	122.2 cd	216.6 cd	16.6 gh
PR04-19^ b^	Unripe	235.6 b	118.9 b	9.1 i	ND c	ND d	47.5 cd	43.3 fg	36.6 hi	28.2 f
	Ripe	484.7 a	161.5 a	8.4 i	ND c	ND d	43.4 de	34.1 g	22.5 i	17.1 gh
PR04-32^ b^	Unripe	48.1 fg	28.9 fg	83.3 h	ND c	ND d	101.3 a	167.8 ab	199.2 d	96.6 a
	Ripe	70.4 ef	33.2 ef	73.5 h	ND c	ND d	97.1 a	154.7 abcd	194.0 d	90.3 a
PR04-35^ b^	Unripe	129.3 d	95.7 c	269.1 de	21.6 a	109.5 a	34.4 g	184.5 a	270.2 b	40.3 de
	Ripe	159.9 cd	95.4 c	284.5 d	22.4 a	117.3 a	38.8 ef	133.7 bcd	328.6 a	52.3 c

**Table:** 

Cultivar/Selection	Stage of Maturity	Quercetin-3-*O*-rutinoside	Quercetin-3-*O*-glucoside	Quercetin-3-*O*-arabinoside	Quercetin-3-*O*-rhamnoside	Quercetin-3-*O*-xyloside	Quercetin pentosyl-hexoside	Quercetin pentosyl-pentoside	Quercetin-acetylhexoside
Sun Breeze	Unripe	NQ i	8.9 j	10.1 j	3.9 fgh	2.3 j	4.7 de	2.6 cd	ND e
	Ripe	NQ i	13.3 ij	12.7 j	5.9 defgh	3.0 ghij	4.8 de	2.4 de	0.9 de
African Delight	Unripe	65.5 c	114.3 b	15.9 ij	3.6 gh	2.7 hij	8.7 bc	0.9 fg	20.5 a
	Ripe	70.2 c	129.4 a	11.7 j	3.3 h	2.6 ij	8.6 bc	1.4 f	18.3 ab
Laetitia	Unripe	49.2 d	40.6 ef	28.8 defgh	9.5 bc	4.6 defg	4.9 de	1.7 ef	22.3 a
	Ripe	62.6 c	69.8 d	26.3 fghi	8.1 bcde	4.3 defghi	4.5 e	1.4 f	21.8 a
Ruby Crunch	Unripe	82.8 b	51.1 e	40.7 bc	10.0 bc	6.7 bc	6.0 d	NQ h	4.4 de
	Ripe	92.9 a	87.8 c	25.2 ghi	6.8 cdefg	4.4 defgh	4.5 e	NQ h	3.0 de
Ruby Red	Unripe	15.0 fgh	20.4 hi	44.3 ab	15.4 a	7.5 ab	ND f	4.2 ab	12.6 c
	Ripe	28.8 e	93.0 c	52.7 a	17.0 a	9.2 a	ND f	4.2 a	14.1 bc
Sapphire	Unripe	20.7 efg	65.6 d	38.9 bcde	8.3 bcde	5.8 bcd	7.6 c	3.4 bc	12.4 c
	Ripe	23.3 ef	65.8 d	39.9 bcd	8.4 bcd	6.0 bcd	7.4 c	3.4 abc	11.9 c
PR03-34 ^b^	Unripe	10.6 h	22.4 ghi	19.9 hij	4.9 efgh	3.3 fghij	5.9 d	3.0 cd	4.8 de
	Ripe	11.7 gh	25.2 gh	20.3 hij	5.0 efgh	3.3 efghij	5.6 de	2.8 cd	5.2 d
PR04-19 ^b^	Unripe	18.7 fgh	33.0 fg	36.7 bcdef	8.0 bcde	5.3 cd	10.2 a	1.1 fg	11.6 c
	Ripe	22.4 ef	48.1 e	34.0 bcdefg	7.2 cdef	5.2 cde	9.3 ab	0.4 gh	10.1 c
PR04-32 ^b^	Unripe	9.4 hi	13.8 hij	18.2 hij	3.9 fgh	2.9 ghij	ND f	3.4 abc	5.1 d
	Ripe	9.8 h	13.6 ij	19.3 hij	3.7 gh	2.9 ghij	ND f	3.3 cd	4.5 de
PR04-35 ^b^	Unripe	12.7 gh	15.1 hij	28.5 efgh	10.0 bc	5.0 cdef	ND f	3.1 cd	5.1 d
	Ripe	11.8 gh	15.1 hij	32.1 cdefg	11.1 b	5.4 cd	ND f	3.1 cd	4.9 d

^a^ Values represent averages of plums from three trees (5 fruits per tree); different letters in the same column indicate a statistically significant difference (*p* < 0.05); ^b^ selection numbers for plums without cultivar names currently in evaluation trials; ND, not detected; NQ, not quantified due to low concentration or co-elution.

The TAC and TPC of various *P. salicina* and *P. domestica* plum cultivars grown outside South Africa have been shown to vary considerably between cultivars and selections [12,13,14,15,22]. Similarly, in the current study, large variation was observed between South African cultivars and selections (Table 4). Values for South African cultivars and selections were in the high range compared to literature values when recalculated to the same units. It was expected that Sun Breeze, a yellow-skinned and -fleshed cultivar, would display lower TAC values and TPC relative to the other cultivars and selections due to the absence of anthocyanins, which make up a large part of the total polyphenols of other plum varieties (Table 5). Red fruits also display high TPC and TAC values compared with other fruits (e.g., [4,5,22]). Sun Breeze had among the lowest TAC and TPC values, but these values were not significantly lower than those of Laetitia, African Delight, Sapphire and PR04-19 in most cases. Previous studies on plums (*P. domestica*) showed that plums with yellow and red skins displayed lower TAC values and TPC than plums with blue or purple skin [13]. The a*-value of the flesh was positively correlated (*p* < 0.01) with TAC determined using the ABTS^•+^, DPPH^•^ and FRAP assays and TPC indicating that red-fleshed plums tend to have higher TAC and TPC, although this was not the case for all cultivars and selections. African Delight (yellow-fleshed) was a notable exception with regard to TAC in the DPPH^•^ assay, showing similar TAC as red-fleshed cultivars and selections, while unripe fruits of some red-fleshed cultivars and selections had relatively low TAC and TPC. These results highlight the fact that fruit varieties with red flesh, which is often marketed as more “healthy” does not necessarily have higher nutraceutical value, in terms of TAC and TPC, than yellow-fleshed varieties of the same species. Different flavonoid sub-classes may, however, differ in their bioavailability and *in vivo* bioactivity. Recently, higher intake of anthocyanin-rich foods was shown to be associated with lower levels of inflammation and improvements in insulin resistance [23], indicating a possible benefit for intake of fruits with higher anthocyanin content.

Ruby Red and PR04-19 plums showed a significant (*p* < 0.05) increase in TAC in the ABTS^•+^, DPPH and FRAP assays from unripe to ripe fruit, while the same trend was observed for TPC of only Ruby Red plums (Table 4). For many other cultivars and selections increases in TAC and TPC from unripe to ripe fruit, although not significant (*p* ≥ 0.05), were also observed. According to the literature, the TAC of plums tends to increase during cold storage and/or ripening. Karaman *et al.* [24] found the TAC of plums (*P. salicina*) to increase during 28 days of storage at 0 °C according to the ABTS^•+^ and FRAP assays. Kevers *et al.* [25] reported that the TAC (ORAC and DPPH^•^ assays) and TPC of plums increased during the initial 15 days of storage at room temperature which can be related to ripening of the fruit, where after a decrease was observed. The same trend was observed for the TPC. Mihalache Arion *et al.* [26] also observed an increase in TAC (DPPH^•^ and ORAC assays) and TPC in autumn plum varieties after 10 days of storage at 4 °C. Usenik *et al.* [18] reported no significant changes in TPC during ripening of *P. domestica* plums.

Individual phenolic compounds were identified previously by comparing the retention times and UV-Vis spectra of peaks to those of authentic reference standards where available. Compounds for which no authentic reference standards were available were tentatively identified based on UV-Vis, retention time and mass spectrometric data in one sample of each cultivar/selection under investigation [21].

Cyanidin-3-*O*-glucoside and -rutinoside are some of the major polyphenols in plums with red skin and/or flesh [13,16]. Cyanidin-3-*O*-glucoside was the predominant anthocyanin in South African plums (Table 5). The cyanidin-3-*O*-glucoside content of ripe PR04-19 and Ruby Red fruits was substantially higher (*p* < 0.05) than that of the other cultivars and selections. PR04-19 (ripe) had a significantly higher cyanidin-3-*O*-rutinoside content (161.5 mg/kg fresh weight (FW)) than other cultivars. Ripe red-fleshed plums tended to have higher anthocyanin content than the ripe red-skinned and yellow-fleshed plums. Notable, however, was ripe PR04-32 fruits, which had relatively low anthocyanin content among the red-fleshed plums. Similar results have been obtained previously for peach and plum genotypes [12]. Both the cyanidin-3-*O*-glucoside and -rutinoside contents of Ruby Red and PR04-19 were substantially (*p* < 0.05) higher for ripe fruits compared to unripe fruits, while moderately higher (*p* < 0.05) values were observed for cyanidin-3-*O*-glucoside content of all cultivars and selections, except Sun Breeze, PR04-32 and PR04-35, and the cyanidin-3-*O*-rutinoside content of Sapphire and Ruby Crunch. This can be explained by the accumulation of anthocyanins during ripening as shown in plums [18,27] and strawberries [28]. The accumulation of anthocyanins in plums is related to darker flesh and skin colour (lower flesh and skin L* value) as observed in some cultivars and selections.

Neochlorogenic acid, a hydroxycinnamic acid, is one of the predominant polyphenols in plums [13], with the highest quantity in the current study present in unripe Laetitia fruits (434.8 mg/kg FW; unripe) (Table 5). Ruby Red and African Delight fruits also had very high neochlorogenic acid content of more than 350 mg/kg FW. Neochlorogenic acid was not present in Sapphire fruits, while chlorogenic acid was present only in African Delight, PR03-34 and PR04-35 fruits, and at much lower concentrations (<30 mg/kg FW) than neochlorogenic acid. 3-*O*-*p*-Coumaroylquinic acid was also not ubiquitously present with PR03-34 and PR04-35 fruits having much higher contents (>100 mg/kg FW) than other cultivars and selections (not detected - 23.4 mg/kg FW). The hydroxycinnamic acid content was generally not affected by maturity stage, except that the neochlorogenic acid content of ripe Laetitia plums were lower than that of the unripe fruits.

Plum fruits also contained high levels of flavan-3-ols (Table 5), namely (+)-catechin, (−)-epicatechin, procyanidin B1 and procyanidin B2, with total flavan-3-ol contents between 100 and 600 mg/kg FW. PR04-32 had significantly (*p* < 0.05) higher (−)-epicatechin and procyanidin B2 contents than other cultivars and selections and among the highest (+)-catechin content, while PR04-35 had relatively high (+)-catechin and procyanidin B1 contents. The PCA biplot shows that procyanidin B2 and (−)-epicatechin were closely associated with one another, while procyanidin B1 and (+)-catechin were closely associated. In most cultivars or selections procyanidin B1 and (+)-catechin were present at higher levels than procyanidin B2 and (−)-epicatechin, except for Sapphire, Ruby Red and PR04-19 which had similar levels of these four compounds. Stage of maturity generally did not affect the individual flavan-3-ol contents. However, ripe PR04-35 plums contained significantly (*p* < 0.05) more (−)-epicatechin, procyanidin B1 and procyanidin B2 than the unripe fruits, while the opposite trend was observed for procyanidin B2 content of Ruby Red plums.

In the flavonol group, quercetin-3-*O*-glucoside was predominant in most cultivars and selections, with African Delight having a significantly (*p* < 0.05) higher content than the other cultivars and selections (Table 5). Ruby Crunch and Ruby Red had the highest (*p* < 0.05) quercetin-3-*O*-rutinoside and -rhamnoside content, respectively. The quercetin-3-*O*-glucoside content was significantly (*p* < 0.05) higher in ripe fruits of African Delight, Laetitia, Ruby Red, Ruby Crunch and PR04-19 compared to unripe fruits. However, the content of other flavonol compounds were similar for both ripe and unripe fruits of most cultivars and selections with a few exceptions. Ozturk *et al.* [29] found that the quercetin-3-*O*-rutinoside content of Black Amber (*P. salicina*) plums increased after 28 days at 0 °C storage. However, flavonol aglycones (quercetin and kaempferol) decreased during the same storage period. Olsson *et al.* [30] found that the quercetin content of strawberries increased with ripening, as well as cold storage at 4 °C.

Generally, large variation in the phenolic composition of the different South African plum cultivars and selections was observed. This was to be expected as a previous study by Mubarak *et al.* [14], comparing 29 Australian plum selections, found great variation regarding the contents of neochlorogenic acid, chlorogenic acid, quercetin-3-*O*-rutinoside and (-)-epicatechin between selections.

The Pearson’s correlation matrix showed some interesting correlations between TAC and other measured parameters (Table 2). Significant (*p* < 0.05) negative correlations (*r* from −0.376 to −0.545) were observed between the fruit mass and TAC, except for the ORAC assay (*r* = 0.025; *p* ≥ 0.05). A possible reason for the greater TAC of smaller fruit is the higher skin-to-flesh ratio. Phenolic compounds are more concentrated in the skin of plum fruit [31,32] and would thus provide a greater contribution to the TAC of smaller fruit. The total flavan-3-ol content was significantly (*p* < 0.01) correlated (*r* from 0.542 to 0.712) with TAC in all the assays, while the total anthocyanin content significantly (*p* < 0.05) correlated with TAC in the ABTS^•+^ and FRAP assays. No significant (*p* ≥ 0.05) correlations were observed for total flavonols and hydroxycinnamic acid contents with TAC. Flavan-3-ols were shown to contribute greatly to the antioxidant capacity of plum fruits using on-line antioxidant assays [21]. The high TAC displayed by PR04-32 and PR04-35 are likely explained by their relatively high flavan-3-ol content.

The high contribution of flavan-3-ols to the TAC of plum fruits can be explained based on their structural features. The structure of phenolic compounds greatly affects their ability to act as antioxidants. For example, the flavonoids are known to be effective radical scavengers due to presence of the catechol group [33], while hydroxycinnamic acids act as radical scavengers through hydrogen donation from the carboxyl side-chain (as reviewed by El–Seedi *et al.* [34]). Structural features conferring high antioxidant activity to flavan-3-ols include the catechol group on the B-ring and unobstructed 3-OH group. Tsao *et al.* [35] used the FRAP assay to compare antioxidant activity of various phenolic compounds and found that procyanidin B1, procyanidin B2 and (−)-epicatechin exhibited a higher TAC than quercetin-3-*O*-glycosides and chlorogenic acid. Tabart *et al* [36] found that phenolic acids exhibited lower TAC than flavonols, flavan-3-ols and anthocyanins as determined with the ORAC assay, and lower than flavan-3-ols with the DPPH^•^ assay. Differences observed between assays can be attributed to the fact that antioxidants have a multifunctional nature and the assays are based on different mechanisms. The DPPH^•^ and ABTS^•+^ assays are based on the ability of the antioxidant to scavenge a synthetic radical, and the ORAC assay measures the ability of an antioxidant to act against peroxyl radicals [37]. Although the DPPH^•^ and ABTS^•+^ assays are both based on radical scavenging, the DPPH^•^ assay is based on electron transfer, while ABTS^•+^ is based on hydrogen donation [37].

### 2.2. Comparison of Harvest Seasons

**Figure 3:**
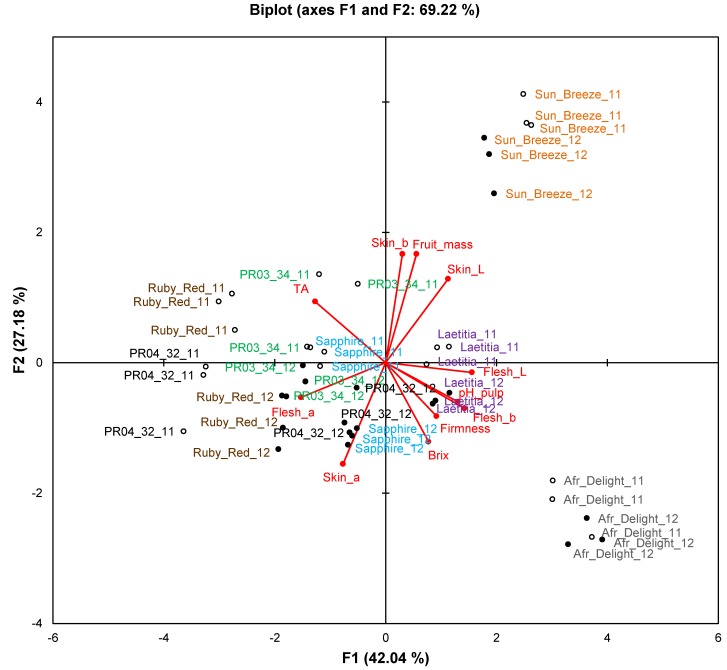
Principal component biplot of fruit attributes of ripe fruits from South African plum (*Prunus salicina* Lindl.) cultivars and selections of two consecutive harvest seasons.

*Abbreviations:* 11, first harvest season (2010/2011; open circles); 12, second harvest season (2011/2012; closed circles); Refer to Figure 1 for other abbreviations.

Only data for ripe fruits will be discussed in this section to compare the two consecutive harvest seasons. The biplot for fruit attributes (Figure 3) explains 69.2% of the variation. Observations formed separate clusters for the respective seasons, indicating that the two seasons differed from one another in terms of fruit attributes. Overlap between seasons was only observed for African Delight. No single fruit attribute was responsible for this phenomenon as evidenced by ANOVA results (Table 6). Fruit mass was generally similar between harvest seasons with only Laetitia, Ruby Red and PR03-34 having a significantly (*p* < 0.05) higher fruit mass in the first harvest season. The pH of Sun Breeze, Sapphire, Ruby Red and PR04-32 fruits were significantly (*p* < 0.05) higher in the second harvest season, while the TA of African Delight and Laetitia was significantly (*p* < 0.05) higher and that of Sapphire, Ruby Red and PR04-32 was significantly (*p* < 0.05) lower in the second harvest season. The TDS of Sun Breeze, Ruby Red and PR04-32 was significantly (*p* < 0.05) higher in the second harvest season. Trends for fruit colour attributes were cultivar-dependent with that of African Delight, Laetitia and Ruby Red not significantly (*p* ≥ 0.05) affected by harvest season.

**Figure 4:**
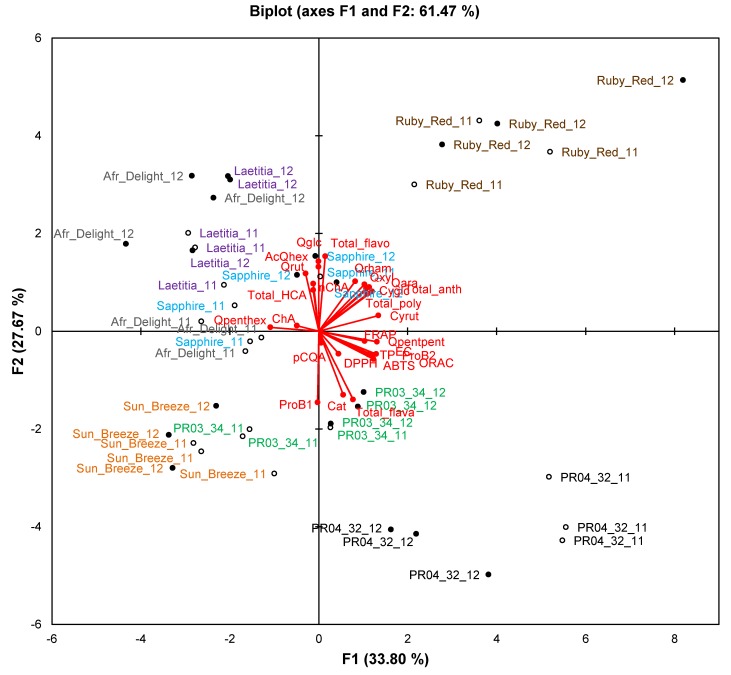
Principal component biplot of phenolic composition and total antioxidant capacity of ripe fruits from South African plum (*Prunus salicina* Lindl.) cultivars and selections of two consecutive harvest seasons.

*Abbreviations:* Refer to Figure 2 and Figure 3.

On the PCA biplot for phenolic composition and TAC (61.5% of variation explained), clear separation between seasons was seen only for African Delight and PR04-32 (Figure 4). Clusters for a specific cultivar retained their general spatial distribution on the biplot, indicating that seasonal effects did not obscure cultivar differences.

Among the TAC assays, the ORAC assay showed no significant (*p* ≥ 0.05) effect for harvest season, while all values obtained using the DPPH^•^ assay in the first harvest season were higher than in the second harvest season (Table 7). TAC values obtained using the ABTS^•+^ and FRAP assays were generally not affected by harvest season except that PR03-34 and PR04-32 showed higher and lower values, respectively, for the second harvest season. The TPC obtained using the Folin-Ciocalteau assay showed a trend similar to that of the ORAC assay.

The phenolic composition of fruits was affected by harvest season, but trends were specific to each cultivar (Table 8). Generally, few significant changes were observed for the anthocyanin content between harvest seasons. The only exceptions were PR03-34, for which cyanidin-3-*O*-glucoside showed a significant increase (*p* < 0.05) from the first to the second season, and PR04-32 for which the content of both anthocyanins decreased significantly (*p* < 0.05). Among the hydroxycinnamic acids, the chlorogenic and 3-*O*-*p*-coumaroylquinic acid contents of PR03-34 were significantly (*p* < 0.05) higher in the second harvest season, while the same trend was observed for neochlorogenic acid content of Ruby Red. The content of some individual flavan-3-ols were significantly (*p* < 0.05) lower in the second harvest season for Sun Breeze ((+)-catechin), African Delight ((−)-epicatechin, (+)-catechin and procyanidin B1), Laetitia ((+)-catechin) and PR04-32 ((−)-epicatechin and (+)-catechin). Overall, the individual flavonol content of cultivars and selections was either unaffected by season, or was significantly (*p* < 0.05) higher in the second harvest season, except in the case of Ruby Red and PR04-32. For both, the quercetin-3-*O*-rutinoside content was significantly (*p* < 0.05) lower in the second harvest season, while the same trend was observed for quercetin-acetylhexoside content of PR04-32.

By comparing two harvest seasons it is evident that season affects the composition of fruit, but that it does not obscure cultivar differences. Due to the diversity of fruit attributes, phenolic content and TAC, specific trends could not be assigned for changes from one season to the next. In a study by Kim *et al.* [20], plum cultivars (*P. domestica*) grown in New York were compared in terms of phenolic composition over two harvest seasons. They showed that the content of various phenolic compounds differs between cultivars, as well as from one harvest season to the following.

**Table 6:** Fruit attributes for ripe fruits from South African plum (*Prunus salicina* Lindl.) cultivars and selections harvested during two consecutive harvest seasons ^a^.

Cultivar/Selection	Harvest Season	Fruit Mass (g)	Firmness (kg)	pH	TA ^b^	TDS (°Brix) ^c^	Skin a *^,d^	Skin b *^,d^	Skin L *^,d^	Flesh a *^,d^	Flesh b*^,d^	Flesh L *^,d^
Sun Breeze	First	113.4 a	3.8 c	3.90 f	22.0 de	12.6 gh	-3.4 f	35.0 a	59.3 a	−1.1 h	19.7 de	48.0 a
	Second	105.9 ab	3.0 cd	3.97 cd	24.5 cd	25.3 cde	-0.5 f	27.3 b	58.4 a	−0.3 h	17.6 ef	41.7 b
African Delight	First	83.4 de	5.4 b	4.24 a	9.9 h	18.6 a	18.0 d	−0.5 f	42.7 c	3.5 fg	25.7 a	49.0 a
	Second	77.8 ef	7.9 a	4.22 a	12.5 g	19.3 a	18.8 d	0.0 ef	42.8 c	2.3 g	27.4 a	51.5 a
Laetitia	First	98.1 bc	0.2 e	3.95 cde	19.1 f	13.2 efgh	20.6 cd	0.8 ef	41.7 c	4.7 f	22.1 bc	48.3 a
	Second	82.9 de	2.7 cd	3.98 bc	22.9 cde	13.7 defg	21.8 c	1.3 ef	41.4 c	4.2 fg	23.0 b	48.3 a
Ruby Red	First	101.3 abc	0.2 e	3.77 h	33.8 a	15.0 c	13.8 e	−2.0 f	29.8 de	18.8 c	10.0 j	27.6 f
	Second	78.1 ef	5.4 b	3.86 g	30.4 b	16.4 b	15.0 e	−1.7 f	29.7 de	20.5 bc	12.2 ij	30.5 ef
Sapphire	First	85.8 de	0.0 e	3.53 i	24.7 c	13.0 fgh	19.3 cd	3.0 e	30.0 de	8.9 e	20.1 cd	41.3 bc
	Second	78.5 ef	2.4 d	4.02 b	20.4 ef	14.1 cdef	19.6 cd	3.1 e	28.0 e	15.6 d	18.5 def	38.1 cd
PR03-34 ^e^	First	93.1 cd	2.8 cd	3.93 def	23.3 cd	12.7 gh	20.1 cd	16.4 c	46.2 b	21.3 b	14.0 hi	33.0 e
	Second	75.0 ef	2.9 cd	3.93 ef	22.5 cde	14.6 cd	25.5 b	14.5 c	42.3 c	20.6 bc	11.6 j	29.1 f
PR04-32 ^e^	First	66.6 f	0.9 e	3.39 j	24.6 cd	12.5 h	28.9 a	10.8 d	32.6 d	25.8 a	15.1 gh	30.2 ef
	Second	75.0 ef	6.3 b	3.97 c	20.7 ef	12.3 h	29.2 a	15.1 c	40.2 c	19.6 bc	16.6 fg	36.6 d

^a^ Values represent averages of plums from three trees (5 fruits per tree); different letters in the same column indicate a statistically significant difference (*p* < 0.05); ^b^ titratable acidity in g malic acid/kg fresh weight; ^c^ total dissolved solids expressed as °Brix; ^d^ skin and flesh colour values using the CIELab scale; ^e^ selection numbers for plums without cultivar names currently in evaluation trials.

## 3. Experimental

### 3.1. Chemicals

Authentic reference standards (purity indicated in brackets) were obtained from Fluka [Sigma-Aldrich, St. Louis, MO, USA; quercetin-3-*O*-glucoside (≥90%), quercetin-3-*O*-rhamnoside (≥97%) and chlorogenic acid (≥95%)], PhytoLab [Vestenbergsgreuth, Germany; quercetin-3-*O*-arabinoside (87%), neochlorogenic acid (98%), procyanidins B1 (94%) and procyanidin B2 (95%)], Sigma-Aldrich [St. Louis, MO, USA; quercetin-3-*O*-rutinoside (≥94%), (+)-catechin (≥98%) and (−)-epicatechin (≥98%)], Extrasynthese [Genay, France; cyanidin-3-*O*-rutinoside (97%)] and Polyphenols Laboratories Sandnes, Norway; cyanidin-3-*O*-glucoside (≥97%) and -galactoside (≥97%). Trifluoroacetic acid (TFA) and acetonitrile (gradient grade for liquid chromatography) were purchased from Sigma-Aldrich. The ABTS [2,2'-azinobis-(3-ethyl-benzothiazoline-6-sulphonic acid)] reagent was supplied by Roche Diagnostics GmbH (Indianapolis, IN, USA). All other reagents were analytical grade and were obtained from Merck Millipore (Darmstadt, Germany) and Sigma-Aldrich. Laboratory grade deionised water was prepared using an Elix (Merck Millipore) water purification system and subsequently subjected to an additional purification step using a Milli-Q academic (Merck Millipore) water purification system to obtain HPLC grade water.

**Table 7:** Total polyphenol content (TPC) ^a^ and total antioxidant capacity (TAC) ^b^ for ripe fruits from South African plum (*Prunus salicina* Lindl.) cultivars and selections harvested during two consecutive harvest seasons ^c^.

Cultivar/Selection	Harvest Season	TPC ^a^	ABTS^•+^^b,d^	DPPH^•^^b,e^	FRAP ^b,f^	ORAC ^b,g^
Sun Breeze	First	2.60 cdef	23.9 def	20.6 c	9.6 ef	38.9 def
	Second	2.32 ef	21.9 f	12.5 h	9.0 f	41.4 def
African Delight	First	2.90 bc	27.5 cd	27.9 a	11.8 bcd	46.8 cde
	Second	2.65 cde	23.8 def	16.4 ef	10.5 def	38.4 ef
Laetitia	First	2.44 def	22.3 f	19.1 cd	9.5 ef	37.1 ef
	Second	2.58 cdef	23.0 ef	12.4 h	9.8 ef	35.4 ef
Ruby Red	First	2.84 bcd	27.5 cd	23.3 b	12.7 ab	51.2 bcd
	Second	2.98 bc	29.7 bc	14.6 fg	12.5 b	55.4 abc
Sapphire	First	2.22 f	21.8 f	20.5 c	9.3 ef	33.9 f
	Second	2.27 ef	25.0 def	13.3 gh	9.7 ef	32.6 f
PR03-34 ^h^	First	2.78 bcd	26.8 cde	22.7 b	10.8 cde	40.7 def
	Second	2.98 bc	33.0 ab	15.5 f	12.3 bc	42.1 def
PR04-32 ^h^	First	3.43 a	35.4 a	27.8 a	14.4 a	67.1 a
	Second	3.16 ab	32.5 ab	17.7 de	12.5 b	62.2 ab

^a^ TPC in g gallic acid equivalents/kg fresh weight as measured using the Folin-Ciocalteau assay; ^b^ TAC in µmol Trolox equivalents/g fresh weight; ^c^ values represent averages of plums from three trees (5 fruits per tree); different letters in the same column indicate a statistically significant difference (*p* < 0.05); ^d^ TAC measured using the ABTS^•+^ scavenging assay; ^e^ TAC measured using the DPPH^•^ scavenging assay; ^f^ TAC measured using the ferric reducing antioxidant power assay; ^g^ TAC measured using the oxygen radical absorbance capacity assay; ^h^ selection numbers for plums without cultivar names currently in evaluation trials.

### 3.2. Sample Collection

Plum fruit (*Prunus salicina* Lindl.) were harvested at commercial ripeness, *i.e.*, during the pre-climacteric phase. The harvest date was determined based on fruit firmness, soluble solids content and colour depending on the cultivar. On the day of harvest, 12 randomly selected plums from each of three trees per cultivar or selection (Table 1) were harvested (Bien Donné, Groot Drakenstein, South Africa; S 33.84, E 18.98). Five fruits from each tree were used for analysis on the day of harvest (unripe). The remaining fruit were subjected to a commonly used commercial cold storage and ripening regime, advancing the fruit to eating ripeness. This involved storage for 10 days at −0.5 °C, followed by 9 days at 7.5 °C, 16 days at −0.5°C, and ripening at 10 °C for 7 days. Five fruits from each tree were used for analysis after cold storage and ripening (ripe).

Table 8Individual phenolic compound content (mg/kg fresh weight) ^a^ for ripe fruits from South African plum (*Prunus salicina* Lindl.) cultivars and selections harvested during two consecutive harvest seasons ^b^.

**Table:** 

Cultivar/Selection	Harvest Year	Cyanidin-3-*O*-glucoside	Cyanidin-3-*O*-rutinoside	Neochlorogenic Acid	Chlorogenic Acid	3-*O-p*-Coumaroyl-quinic Acid	(−)-Epicatechin	(+)-Catechin	Procyanidin B1	Procyanidin B2
Sun Breeze	First	ND d	ND e	216.3 c	ND d	14.6 ef	11.0 efg	98.2 e	211.1 a	14.1 c
	Second	ND d	ND e	212.7 c	ND d	10.7 f	8.4 fg	75.5 fg	204.8 a	17.0 c
African Delight	First	72.3 c	10.0 e	388.9 ab	19.9 a	ND g	21.0 ef	126.5 c	146.5 b	9.3 c
	Second	69.8 c	13.1 e	356.0 b	21.7 a	ND g	5.0 g	67.0 gh	104.4 c	9.9 c
Laetitia	First	51.4 c	15.6 de	334.2 b	ND d	16.3 de	17.0 efg	89.1 ef	105.7 c	8.9 c
	Second	52.5 c	15.3 de	376.3 ab	ND d	15.1 ef	10.5 efg	66.5 gh	99.8 cd	8.6 c
Ruby Red	First	447.0 a	91.4 a	353.7 b	ND d	20.8 cd	59.2 c	57.7 gh	51.6 e	43.5 b
	Second	474.9 a	87.4 a	433.0 a	ND d	23.4 c	57.6 cd	56.4 h	64.9 de	52.6 b
Sapphire	First	208.9 b	43.7 bc	ND e	ND d	ND g	44.1 d	49.9 h	64.6 de	48.2 b
	Second	231.6 b	56.5 b	ND e	ND d	ND g	51.5 cd	60.9 gh	73.5 cde	47.6 b
PR03-34 ^b^	First	92.2 c	38.0 bc	218.1 c	8.8 c	99.6 b	17.0 efg	107.5 de	204.3 a	14.9 c
	Second	232.8 b	55.5 b	255.6 c	11.4 b	115.4 a	22.4 e	122.2 cd	216.6 a	16.6 c
PR04-32 ^b^	First	188.8 b	100.8 a	65.8 d	ND d	ND g	113.1 a	189.3 a	209.0 a	82.1 a
	Second	70.4 c	33.2 cd	73.5 d	ND d	ND g	97.1 b	154.7 b	194.0 a	90.3 a

**Table:** 

Cultivar/Selection	Harvest Year	Quercetin-3-*O*-rutinoside	Quercetin-3-*O*-glucoside	Quercetin-3-*O*-arabinoside	Quercetin-3-*O*-rhamnoside	Quercetin-3-*O*-xyloside	Quercetin pentosyl-hexoside	Quercetin pentosyl-pentoside	Quercetin-acetylhexoside
Sun Breeze	First	NQ g	11.7 e	18.0 efg	5.7 cdef	3.6 def	6.0 cde	3.3 abc	0.4 j
	Second	NQ g	13.3 e	12.7 fg	5.9 cde	3.0 def	4.8 fg	2.4 cd	0.9 ij
African Delight	First	44.7 bc	90.9 b	10.2 g	2.7 f	2.0 f	6.7 bcd	ND f	10.2 ef
	Second	70.2 a	129.4 a	11.8 fg	3.3 ef	2.6 ef	8.6 a	1.4 e	18.3 bc
Laetitia	First	53.6 b	63.5 cd	20.1 defg	4.6 ef	3.3 def	2.8 h	ND f	23.6 a
	Second	64.2 a	69.8 c	26.3 cde	8.1 bcd	4.3 cde	4.5 g	1.4 e	21.8 ab
Ruby Red	First	39.1 c	95.9 b	40.6 b	10.6 b	6.9 b	ND i	3.0 bcd	16.7 cd
	Second	28.8 d	93.0 b	52.7 a	17.0 a	9.2 a	ND i	4.2 a	14.1 de
Sapphire	First	23.2 d	49.8 d	30.5 bc	6.3 cde	4.1 de	7.0 bc	2.7 bcd	10.5 ef
	Second	23.3 d	65.8 c	39.9 b	8.4 bc	6.0 bc	7.4 b	3.4 ab	11.9 ef
PR03-34 ^b^	First	8.4 fg	10.1 e	20.5 cdef	5.1 def	2.9 def	5.9 de	2.3 de	5.6 gh
	Second	11.7 ef	25.2 e	20.3 cdefg	5.0 def	3.3 def	5.6 ef	2.8 bcd	5.2 gh
PR04-32 ^b^	First	20.8 de	24.9 e	29.3 cd	4.7 ef	4.6 cd	ND i	3.4 ab	8.6 fg
	Second	9.8 f	13.6 e	19.3 defg	3.7 ef	2.9 def	ND i	3.3 abc	4.5 hi

^a^ Values represent averages of plums from three trees (5 fruits per tree); different letters in the same column indicate a statistically significant difference (*p* < 0.05); ^b^ selection numbers for plums without cultivar names currently in evaluation trials; ND, not detected; NQ, not quantified due to low concentration or co-elution.

Fruit were analysed for the following fruit attributes using standard techniques: firmness (kg, penetrometer with 11 mm tip), pH (pH meter), titratable acidity (g malic acid/kg fresh weight (FW), automatic titrator) and soluble solids content (°Brix, refractometer). The depitted fruit, including fruit flesh and skin, of each tree were homogenised together with sodium fluoride (added as 1 mL for every 4 g fruit at a concentration of 4 g/L) to prevent enzymatic oxidation of phenolic compounds [32] using a food blender. Aliquots were frozen at ca −20°C until extraction. 

### 3.3. Fruit Colour Analysis

Skin and flesh colour were measured with a CR-400 Konica Minolta Colorimeter (Tokyo, Japan). Four measurements were taken at random positions on the skin for skin colour. A slice of skin and flesh were removed with a knife from the equatorial region on both sides of the suture of each fruit to provide a flat surface for colour measurement (see photo of PR04-32 in Table 1), which entailed three measurements on each exposed flesh area. The colorimeter measured the colour of the sample on the CIELab scale using the 2° observer and C-illuminant. The values measured indicate the lightness (L*), red and green colour (positive and negative a*-values), and the yellow or blue colour (positive and negative b*-values). 

### 3.4. Extraction of Pulp

The frozen plum pulp was defrosted at room temperature and duplicate extracts prepared. For extraction, ca 5.0 g pulp was weighed into a 50 mL screw-cap centrifuge tube and 10 mL methanol added, followed by sonication (Branson 8510, Branson Ultrasonic Corporation, Danbury, CT, USA) for 10 min. The tubes were centrifuged for 10 min at 8000 rpm (*ca.* 6,000 *×g*) using a Biofuge Primo Centrifuge (Thermo Scientific, AEC-Amersham, Johannesburg, South Africa) to separate the solids from the liquid. The supernatant was filtered using a Millex-HV hydrophilic polyvinylidene difluoride (PVDF) 0.45 µm syringe-driven filter (Merck Millipore). Thereafter, 300 µL aliquots of the filtrate were diluted with 1 mL deionised water and frozen at ca −20 °C until analysis.

### 3.5. Determination of Total Polyphenol Content and Total Antioxidant Capacity

The TPC of the plum extracts was determined in triplicate using the Folin-Ciocalteau method modified for use in microplates as described by Arthur *et al.* [38]. The TAC of the extracts was determined in triplicate using the 2,2-diphenyl-1-picrylhydrazyl radical (DPPH^•^) scavenging [38], ABTS^•+^ scavenging [39], Ferric Reducing Antioxidant Power (FRAP) [40] and Oxygen Radical Absorbance Capacity (ORAC) [41] assays. The ABTS^•+^ scavenging and FRAP assays were scaled down to a 200 µL reaction volume and performed in 96-well microplates. The assays were performed using a BioTek SynergyHT microplate reader with Gen5 software (Winooski, VT, USA) for the collection of data. Clear flat-bottomed polystyrene 96-well microplates (Greiner Bio-One, Frickenhausen, Germany) were used for the absorbance assays, while a black plate with a clear flat bottom (Greiner Bio-One) was used for fluorescence measurements (ORAC assay).

### 3.6. Determination of Phenolic Composition Using High-Performance Liquid Chromatography with Diode-Array and Fluorescence Detection (HPLC-DAD-FLD)

The phenolic composition of plum extracts was determined using the validated method described by Venter *et al.* [21]. Analyses were performed using an Agilent 1200 series HPLC (Waldbronn, Germany) consisting of an autosampler, quaternary pump, column thermostat, diode-array detector and fluorescence detector. Chemstation software for LC 3D systems (Agilent) was used for data acquisition and analysis. A Gemini-NX C18 column (3 μm; 110 Å; 150 × 4.6 mm; Phenomenex, Santa Clara, CA, USA) protected by a guard column packed with the same stationary phase (4 × 3.0 mm; Phenomenex) was used. The mobile phases were 0.05% TFA (A) and acetonitrile (B) and the separation was performed at 40 °C and a flow rate of 1 mL/min. The mobile phase gradient was as follows: 0–2 min (3% B), 2–30 min (3%–35% B), 30–31 min (35%–50% B), 31–33 min (50% B), 33–35 min (50%–3% B), 35–45 min (3% B). Hydroxycinnamic acids were quantified at 320 nm, flavonols at 350 nm and anthocyanins at 520 nm. Flavan-3-ols were quantified using a fluorescence detector (λ_ex_ = 275 nm; λ_em_ = 315 nm). Two injection volumes (100 µL and either 40 or 50 µL) were used for samples to ensure accurate quantification of compounds present in small and large concentrations. Calibration curves were set up for the following compounds with the amount injected indicated in brackets: neochlorogenic acid (0.03–1.6 µg injected); chlorogenic acid (0.03–1.5 µg); cyanidin-3-*O*-glucoside (0.07–3.2 µg); cyanidin-3-*O*-rutinoside (0.04–2.0 µg); quercetin-3-*O*-rutinoside and -galactoside (0.01–0.5 µg); quercetin-3-*O*-glucoside, -rhamnoside and -arabinoside (0.02–1.0 µg); (+)-catechin, (−)-epicatechin, procyanidin B1 and procyanidin B2 (0.02–1.0 µg). Compounds were identified previously by comparing the retention times and UV-Vis spectra of peaks to those of authentic reference standards where available. Compounds for which no authentic reference standards were available were tentatively identified based on UV-Vis, retention time and mass spectrometric data in one sample of each cultivar/selection under investigation [25]. Quercetin-3-*O*-xyloside, quercetin pentosyl-pentoside and quercetin-acetylhexoside were quantified using quercetin-3-*O*-glucoside equivalents, while 3-*O-p*-coumaroylquinic acid was quantified using neochlorogenic acid equivalents. Phenolic composition data were expressed as mg compound/kg FW.

### 3.7. Statistical Analysis

The experimental design for statistical analysis was treated as a split plot. Plum cultivars were regarded as main plot treatments, while the different maturity stages (unripe and ripe) or harvest seasons were regarded as split plot factors. This design was chosen as fruits from the same tree were used at different maturity stages and in different harvest seasons. For the main plot three trees of each of the cultivars and selections were used as replicates, forming a randomised main plot design.

SAS statistical software (SAS^®^, Version 9.2; SAS Institute Inc., Cary, NC, USA) was used for the univariate analysis of variance (ANOVA) tests and determination of Pearson’s correlation coefficients (*r*). This was done on all variables assessed during the study and the General Linear Models (GLM) procedure was applied. In order to compare the sample treatment means Student’s t-test was used, with the least significant difference calculated at 5%. The Shapiro-Wilk test was used to test for normality and a 5% probability level was regarded as significant. 

Principal Component Analysis (PCA) was performed using XLSTAT software (Version 7.5.2, Addinsoft, New York, NY, USA) and used to evaluate relationships between sample attributes and cultivars and selections as described in Næs *et al.* [42].

## 4. Conclusions

South African plums were shown to have high nutraceutical value. The phenolic composition of yellow- and red-fleshed South African plum cultivars and selections varied greatly. Plums with red skin and flesh tended to have higher TPC and TAC than yellow-fleshed (yellow or red skin) plums, although this was not always the case. The red-fleshed selections PR04-32 and PR04-35 have especially high nutraceutical value, *i.e.*, higher TAC and TPC than some commercial cultivars, although this was due to a high flavan-3-ol content and not linked to their flesh colour. These selections would be good choices to pursue in further evaluation trials to determine their suitability for release as new cultivars with high nutraceutical value. The anthocyanin content of plums generally increased after a commercial cold storage regime followed by ripening, while changes for other phenolic compounds were cultivar-dependent. These changes in phenolic profile led to an increase in TAC in only a few cases. Season affected the phenolic composition, TPC and TAC of plums, although cultivar differences were not obscured.

The current study provided insight into the phenolic composition and antioxidant activity of South African plum cultivars and selections and the effect of a commercial cold storage and ripening regime, topics on which there is limited knowledge. It is believed these findings could benefit current plum breeding programs, as well as future studies on South African plums.

## Supplementary Materials

Supplementary materials can be accessed at: http://www.mdpi.com/1420-3049/19/3/3084/s1.
