# Supplementary material for: Nutraceutical Value of Yellow- and Red-Fleshed South African Plums (Prunus salicina Lindl.): Evaluation of Total Antioxidant Capacity and Phenolic Composition
Source: Molecules. 2014 Mar 11;19(3):3084–109. doi: 10.3390/molecules19033084 (PMC6271285; doi:10.3390/molecules19033084)
Supplement: Supplementary file 1 [file molecules-19-03084-s001.pdf]

Supplementary Materials

Table S1. Fruit attributes of unripe and ripe fruits from South African plum (*Prunus salicina* Lindl.) cultivars and selections of the first harvest season [a].

Cultivar/ Selection	Stage of Maturity	Fruit Mass (g)	Firmness (kg)	pH	TA [b]	TDS (°Brix) [c]	Skin a *,[d]	Skin b *,[d]	Skin L *,[d]	Flesh a *,[d]	Flesh b *,[d]	Flesh L *,[d]
Sun Breeze	Unripe	124.0 a	4.8 c	3.94 b	25.1 d	13.6 defg	−9.9 i	32.4 a	57.0 a	−5.0 l	23.7 c	49.4 bc
	Ripe	113.4 b	3.8 cd	3.90 bcd	22.0 e	12.6 g	−3.4 h	35.0 a	59.3 a	−1.1 k	19.7 e	48.0 c
African Delight	Unripe	89.5 fg	8.7 a	4.24 a	12.6 g	19.7 a	23.5 bc	4.2 de	48.4 b	1.2 ij	28.2 a	51.2 b
	Ripe	83.4 g	5.4 bc	4.21 a	9.9 h	18.6 a	18.0 de	−0.5 fg	42.7 c	3.5 gh	25.7 b	49.0 bc
Laetitia	Unripe	97.6 def	5.2 bc	3.85 cd	29.7 c	14.0 cde	23.3 bc	6.1 d	46.4 b	2.0 hi	28.1 a	53.9 a
	Ripe	98.1 def	0.2 e	3.95 b	19.1 f	13.2 defg	20.6 bcd	0.8 efg	41.7 c	4.7 g	22.1 d	48.3 c
Ruby Red	Unripe	109.6 bc	3.8 cd	3.66 f	43.8 a	16.5 b	17.7 de	3.3 de	37.6 d	12.3 e	20.0 e	40.5 d
	Ripe	101.3 cde	0.2 e	3.77 e	33.8 b	15.0 c	13.8 fg	−2.0 g	29.8 f	18.8 d	10.0 h	27.6 g
Sapphire	Unripe	105.8 bcd	5.0 c	3.34 h	29.5 c	14.2 cd	15.2 ef	5.7 d	36.5 d	−0.1 jk	25.9 b	47.2 c
	Ripe	85.8 g	0.0 e	3.54 g	24.7 d	13.0 efg	19.3 d	2.9 def	30.0 ef	8.9 f	20.1 e	41.3 d
PR03-34 [e]	Unripe	92.2 efg	5.1 c	3.85 cde	29.5 c	13.7 def	11.3 g	16.9 b	47.5 b	24.0 b	17.4 f	34.9 ef
	Ripe	93.1 efg	2.8 d	3.93 bc	23.3 de	12.7 fg	20.1 cd	16.4 b	46.2 b	21.3 c	14.0 g	33.0 f
PR04-32 [e]	Unripe	71.9 h	6.9 ab	3.82 de	28.8 c	13.3 defg	23.8 b	11.9 c	35.7 d	25.8 a	19.1 e	37.0 e
	Ripe	66.6 h	0.9 e	3.39 h	24.6 d	12.5 g	28.9 a	10.8 c	32.6 e	25.8 a	15.1 g	30.2 g

[a] Values represent averages of plums from three trees (5 fruits per tree); different letters in the same column indicate a statistically significant difference ($p < 0.05$); [b] titratable acidity in g malic acid/kg fresh weight; [c] total dissolved solids expressed as °Brix; [d] skin and flesh colour values using the CIELab scale; [e] selection numbers for plums without cultivar names currently in evaluation trials.

Table S2. Total polyphenol content (TPC) [a] and total antioxidant capacity (TAC) [b] of unripe and ripe fruits from South African plum (*Prunus salicina* Lindl.) cultivars and selections of the first harvest season [c].

Cultivar/Selection	Stage of Maturity	TPC [a]	ABTS$^{•+}$ [b,d]	DPPH$^{•}$ [b,e]	FRAP [b,f]	ORAC [b,g]
Sun Breeze	Unripe	2.43 de	23.0 cd	19.8 ef	9.2 f	38.5 cde
	Ripe	2.60 bcd	23.9 bcd	20.6 def	9.6 def	38.9 cde
African Delight	Unripe	2.71 bcd	24.9 bcd	24.7 b	10.9 cd	41.5 cd
	Ripe	2.90 b	27.5 b	27.9 a	11.8 bc	46.8 bc
Laetitia	Unripe	2.57 cd	23.0 cd	19.6 ef	9.9 def	37.1 de
	Ripe	2.44 de	22.3 de	19.1 f	9.5 def	37.1 de
Ruby Red	Unripe	2.43 de	22.2 de	18.5 f	10.1 def	47.6 bc
	Ripe	2.84 bc	27.5 b	23.3 bc	12.7 b	51.2 b
Sapphire	Unripe	1.87 f	18.0 e	16.2 g	7.6 g	31.1 e
	Ripe	2.22 e	21.8 de	20.5 ef	9.3 ef	33.9 de
PR03-34 [h]	Unripe	2.77 bc	22.4 cd	21.8 cde	10.9 cd	38.8 cde
	Ripe	2.78 bc	26.8 bc	22.7 bcd	10.8 cde	40.7 cd
PR04-32 [h]	Unripe	3.49 a	36.3 a	27.5 a	14.7 a	69.0 a
	Ripe	3.43 a	35.4 a	27.8 a	14.4 a	67.1 a

[a] TPC in g gallic acid equivalents/kg fresh weight as measured using the Folin-Ciocalteau assay; [b] TAC in μmol Trolox equivalents/g fresh weight; [c] values represent averages of plums from three trees (5 fruits per tree); different letters in the same column indicate a statistically significant difference ($p < 0.05$); [d] TAC measured using the ABTS$^{•+}$ scavenging assay; [e] TAC measured using the DPPH$^{•}$ scavenging assay; [f] TAC measured using the ferric reducing antioxidant power assay; [g] TAC measured using the oxygen radical absorbance capacity assay; [h] selection numbers for plums without cultivar names currently in evaluation trials.

Table S3. Individual phenolic compound content (mg/kg fresh weight) of unripe and ripe fruits from South African plum (*Prunus salicina* Lindl.) cultivars and selections of the first harvest season [a].

Cultivar/ Selection	Stage of Maturity	Cyanidin-3-*O*-glucoside	Cyanidin-3-*O*-rutinoside	Neochlorogenic acid	Chlorogenic acid	3-*O*-*p*-Coumaroyl-quinic acid	(−)-Epicatechin	(+)-Catechin	Procyanidin B1	Procyanidin B2
Sun Breeze	Unripe	ND f	ND f	210.1 d	ND c	14.4 c	11.8 fg	104.9 d	216.7 a	14.6 de
	Ripe	ND f	ND f	216.3 d	ND c	14.6 c	11.0 g	98.2 de	211.1 a	14.1 def
African Delight	Unripe	23.4 def	5.1 f	386.6 ab	19.7 a	ND d	21.6 e	132.9 c	145.7 c	9.3 ef
	Ripe	72.33 de	10.0 f	388.9 ab	19.9 a	ND d	21.0 e	126.5 c	146.5 c	9.3 ef
Laetitia	Unripe	13.0 ef	6.1 f	395.8 a	ND c	18.8 b	16.8 ef	102.0 d	119.0 cd	8.9 f
	Ripe	51.4 def	15.6 ef	334.2 c	ND c	16.3 c	17.0 ef	89.1 e	105.7 d	8.9 f
Ruby Red	Unripe	80.4 de	45.8 bc	349.5 bc	ND c	19.6 b	63.8 c	70.3 f	62.7 e	49.1 b
	Ripe	447.0 a	91.4 a	353.7 abc	ND c	20.8 b	59.3 c	57.7 g	51.6 e	43.5 c
Sapphire	Unripe	48.6 def	18.1 def	ND f	ND c	ND d	43.3 d	58.3 g	72.2 e	51.3 b
	Ripe	208.9 b	43.7 bc	ND f	ND c	ND d	44.1 d	49.9 g	64.6 e	48.2 bc
PR03-34 [b]	Unripe	82.6 de	32.0 cde	178.9 d	8.4 b	98.1 a	16.4 efg	105.7 d	180.0 b	15.4 d
	Ripe	92.2 cd	38.0 cd	218.1 d	8.8 b	99.6 a	17.0 ef	107.5 d	204.3 ab	14.9 d
PR04-32 [b]	Unripe	153.8 bc	61.4 b	69.0 e	ND c	ND d	105.8 b	203.6 a	211.9 a	87.0 a
	Ripe	188.7 b	100.8 a	65.8 e	ND c	ND d	113.1 a	189.3 b	209.0 ab	82.1 a

Table S3. *Cont.*

Cultivar/ Selection	Stage of Maturity	Quercetin-3-*O*-rutinoside	Quercetin-3-*O*-glucoside	Quercetin-3-*O*-arabinoside	Quercetin-3-*O*-rhamnoside	Quercetin-3-*O*-xyloside	Quercetin pentosyl-hexoside	Quercetin pentosyl-pentoside	Quercetin-acetylhexoside
Sun Breeze	Unripe	NQ g	10.0 i	16.3 efg	4.9 bc	2.8 cd	6.0 c	3.3 ab	1.2 ef
	Ripe	NQ g	11.7 hi	18.0 efg	5.7 bc	3.6 cd	6.0 c	3.3 ab	0.4 f
African Delight	Unripe	39.9 bc	69.8 b	9.3 g	1.7 d	0.9 e	6.2 bc	ND f	9.8 c
	Ripe	44.7 ab	90.9 a	10.2 fg	2.7 cd	2.0 de	6.7 abc	ND f	10.2 c
Laetitia	Unripe	57.1 a	42.8 de	23.0 cde	5.4 bc	3.4 cd	3.2 d	ND f	26.3 a
	Ripe	53.6 a	63.5 bc	20.1 def	4.6 bcd	3.3 cd	2.8 d	ND f	23.6 a
Ruby Red	Unripe	29.9 cd	38.5 def	38.7 ab	11.5 a	6.4 ab	ND e	3.0 abc	18.3 b
	Ripe	39.1 bc	95.9 a	40.6 a	10.6 a	6.9 a	ND e	2.9 bc	16.7 b
Sapphire	Unripe	19.6 def	29.6 efg	33.9 ab	6.9 b	4.0 c	7.5 a	2.7 cd	11.4 c
	Ripe	23.2 de	49.8 cd	30.5 abc	6.3 b	4.1 c	7.0 ab	2.7 cd	10.5 c
PR03-34 [b]	Unripe	8.1 fg	9.8 i	19.6 def	5.2 bc	2.8 cd	5.8 c	2.2 e	5.1 de
	Ripe	8.5 fg	10.1 i	20.5 cde	5.1 bc	2.8 cd	5.9 c	2.3 de	5.6 d
PR04-32 [b]	Unripe	15.9 ef	22.5 ghi	28.8 bcd	4.9 bc	3.7 cd	ND e	3.1 abc	8.3 cd
	Ripe	20.8 def	24.9 fgh	29.3 bcd	4.7 bcd	4.6 bc	ND e	3.4 a	8.6 cd

[a] Values represent averages of plums from three trees (5 fruits per tree); different letters in the same column indicate a statistically significant difference ($p < 0.05$);
[b] selection numbers for plums without cultivar names currently in evaluation trials; ND, not detected; NQ, not quantified due to low concentration or co-elution.
